# Comparative study of accompaniment programs for undergraduate degree students in Spanish universities

**DOI:** 10.3389/fpsyg.2023.1165232

**Published:** 2023-06-23

**Authors:** Susana García-Cardo, Marián Queiruga-Dios, Araceli Queiruga-Dios

**Affiliations:** ^1^Doctoral Programme Education in the Knowledge Society, Universidad de Salamanca, Salamanca, Spain; ^2^Instituto de Acompañamiento, Facultad de Comunicación, Universidad Francisco de Vitoria, Pozuelo de Alarcón, Madrid, Spain; ^3^Higher Technical School of Industrial Engineering, Universidad de Salamanca, Salamanca, Spain

**Keywords:** mentoring, accompaniment, mentor, Spanish universities, undergraduate degree students

## Abstract

**Introduction:**

In the last decade, higher education has undergone a transformation in different areas. The most recent and impactful one may have been the need to keep it updated during the COVID-19 pandemic and to be able to teach remotely and affect university life as little as possible. Another significant change is the emergence of personal attention, accompaniment, or mentoring programs, which have become the prevalent leitmotif in many universities.

**Methods:**

This study compares the different programs at 60 Spanish universities. The relevant information collected during this research is related to the existence of an accompaniment program, and in this program, which plays the role of mentor, or what year is it for. Other information collected from the search is related to the type of mentoring programs, whether they are regulated, have a formal program, or are linked to specific courses. Finally, the assessment procedures are also indicated in case any evaluation is used. After the analysis developed during this research, the mentor-mentee program implemented at the Francisco de Vitoria University is detailed, highlighting differences from other programs, its advantages, and students' benefits.

**Results:**

The number of accompaniment and mentoring programs offered by Spanish universities continues to rise. In Spanish universities, some accompaniment and mentoring programs offer different and specific mentoring activities designed to enhance and further the kind of education and preparation institutions of higher learning should ideally provide. Accompaniment processes generally have a longer duration in private universities than in public universities, offering a wider range of programs for both current and incoming students and those with specific needs, such as international students.

**Discussion:**

The authors found that not many studies have highlighted the value of the accompaniment, and even fewer have conducted comparative analyses of the diverse realities across various universities. Mentoring programs will have the potential to be part of a university's strategy to help students succeed when the shortcomings of mentoring programs. This study opens new avenues for research into the ideal profile of mentors to best accompany university students.

## 1. Introduction

The origin of the word *mentor* appears for the first time in Homer's “Odyssey”. Mentor was a character who helped Ulysses in his absence when he left for the long Trojan campaign. Mentor's duties also included the education of Telemachus, the hero's son. According to Greek mythology, the goddess Athena used the face of Mentor when she wanted to communicate with Telemachus during his journey in the search of his father. Thus, a mentor refers to a counselor who can guide and provide wise and prudent advice.

### 1.1. Mentoring definition

If we refer to the dictionary of the Royal Spanish Academy, to mentor is defined as “to advise or guide.” However, no Spanish verb represents the mentoring process and activities. Sometimes the verb derived from the word *mentor* is used in Spanish, but it does not exist as such in the Spanish language dictionary. Similarly, no word in that dictionary expresses the subject receiving the action of the mentor, i.e., a mentee or a protégé.

In Vance and Olson (1998), the book “*The Mentor Connection in Nursing*” was published about the use of qualified mentors in healthcare careers. This was the first time the term was used as a professional improvement methodology, and since then, mentoring has become a fundamental tool for employees' development in an organization. It has been applied to the business environment, where it promotes workers' improvement with the help of more experienced coworkers.

Bozeman and Feeney (2007) provided a definition that is still used in contemporary times. They proposed a chronology of how different authors approximate a definition without succeeding in it. This is their proposal:

“*A process for the informal transmission of knowledge, social capital and psycho-social support perceived by the recipient as relevant to work, career or professional development; mentoring involves informal communication, usually face-to-face and over a sustainable period of time between a person who is perceived to possess more relevant knowledge, wisdom or experience (the mentor) and a person who is perceived to have less (the protégé)*” (p. 17).

However, Kram (1983) identified the following four moments or phases that occur in a mentor-mentee encounter: initiation (the process starts with the mentee–mentor match), cultivation (the most intensive phase, where the mentoring process is performed), separation (this is the phrase related to the personal and psychological change when the mentee's autonomy increases), and redefinition (transition is integral to the mentoring cycle) (Mullen and Klimaitis, 2021). Although Kram and Isabella (1985) did not provide an exact definition, Kram and Isabella (1985) pointed out that mentoring involves an intense relationship in which an older or more experienced person (the mentor) provides two functions to a younger person (the mentee): one is advice on how to develop professionally and the other is personal support, especially psychosocial.

Bozeman and Feeney (2007) and Ghosh (2013) shared a table of the evolution of the mentoring concept over the years. Those definitions have several common aspects and fewer differences. Thus, a mentor is a more senior, skilled, and experienced individual, while the mentee is usually a junior, less experienced person. For these authors, the mentoring relationship involves members of unequal status or peers. It is an intense, long-term process that includes sponsorship and coaching, facilitates exposure and visibility, and offers challenging work or protection. It provides a role model in terms of support and direction. Moreover, the mentor may or may not belong to the same organization, and it involves mutual commitment and accomplishment by sharing values, knowledge, experience, and so on. Finally, it facilitates professional development and career progress through teaching, counseling, psychological support, protection, and sponsorship (Jiang et al., 2022).

In general, all authors, starting with Kram's four moments, refer to mentorship as a relationship between two individuals, almost always of different statuses. They suppose a long-term relationship where life and professional experiences intertwine, where one takes precedence over the other. Primarily focused on the early years of the mentee's career, these authors emphasize the objective of advancing in the professional sphere.

Notably, Noe (1988) highlighted the mentor's role as a role model for young people, guiding them toward their future in a professional trajectory. Ragins et al. (2000) emphasized that a mentor does not always have to be part of the same organization as the mentee or protégé. Hunt and Michael (1983) defined the mentor as someone with experience and knowledge committed to promoting their protégé in the professional world.

Scandura and Schriesheim (1994) highlighted two aspects of this relationship: it is a transformative activity and requires a mutual commitment from both. In this relationship, there is an exchange of values, knowledge, and experience. Furthermore, mentoring can even provide psychological support (Zey, 1984).

According to Díaz-López (2022), mentoring is a privileged opportunity to support students in navigating the back-and-forth between their experiences and their understanding of their current situation and environment. In the presence of their mentor, the mentee creates a reflective space for themselves, contemplating their own experiences, the consequences of their actions, and how these outcomes prompt them to question their beliefs and make decisions that will impact their daily lives when they return to their experiences.

Therefore, it is evident that mentoring cannot be a mere relationship where the intention is the promotion of the accompanied person. Díaz-López (2022) emphasized that mentoring is a relationship of trust with the mentee in an environment of trust, consistency, and the mentor's ability to provide guidance through personal experiences. Confidentiality, attentive listening, moments of silence, and freedom are relevant factors for the mentee, who assumes the role of the protagonist. Conversely, the mentor assumes a supportive role, fostering an atmosphere of respect and acceptance.

### 1.2. Mentoring in higher education

In the higher education context, during the educational accompaniment process, the mentor cannot simply be a professional of a higher rank with extensive experience; however, the mentor should be a trainer specializing in formative accompaniment. Thus, a mentor must possess theoretical solidity, high technical preparation, a genuine commitment to the mentee, constant internal motivation, and an unwavering dedication to their mission while remaining adaptable to various circumstances. Mentoring requires authentic individuals who can serve as authentic life teachers and be witnesses to their mentees, guiding them along their personal journey of growth and fulfillment. This journey, marked by both successes and challenges, is an ongoing and never-ending process (Díaz-López, 2022).

Several studies have been published on the effectiveness of mentoring and peer tutoring. In the context of medical education, peer-assisted learning has a long tradition and significant effectiveness (Topping, 1996; Guraya and Abdalla, 2020).

In Spain, the current Organic Law of the University System (LOSU in Spanish) outlines several rights for students concerning their academic education. These rights encompass access to a tutorial and advice system, psycho-pedagogical guidance, and support for mental and emotional health care. Additionally, students are entitled to receive guidance and information regarding activities that affect them, as well as access to a guidance service that assists them in designing their educational trajectory and facilitates their social and professional integration. Furthermore, students are eligible for academic recognition for their involvement in university mentoring programs, service-learning initiatives, student representation, solidarity activities, and more.

González Alfaya (2008) developed a comparative study about tutoring at the University of Santiago de Compostela (Spain) and the Università degli Studi di Torino (Italy). However, is it related to tutoring, not mentoring? Even so, the author refers to the Spanish model and the different types of mentoring. It emphasizes that the fundamental objective is to advise, train, inform, and help the student navigate their university life.

Pantoja-Vallejo et al. (2022) made a comparison between a Spanish university (the University of Jaén) and an Argentinian one (Argentine University). The authors highlighted the scarce studies carried out on mentoring and tutoring. They developed a study with students who participated in a tutorial action plan. The study emphasized the positive evaluation provided by all students who participated in the plan. They agreed with the initiative of the university, and they considered that it had had a notable impact on their training. A brief study of Spanish universities was developed by Queiruga-Dios et al. (2023), and they found that the mentoring program between university teachers and first-year students is the less common program.

Mentoring is more than tutoring. A tutor trains and helps students in their academic studies and cares for and guides them in an evaluative and supervisory role. In tutoring, the process is shared by the tutor and the tutee. However, in the case of mentoring, the mentee is responsible of his/her own personal growth. The mentor orients, enlightens, and provides advice but is not responsible for the results of the process. Despite these distinctions, the difference between tutors and mentors has become increasingly blurred over time (Soto-Lillo and Quiroga-Lobos, 2021).

The specific features of mentoring in higher education may be defined as follows (Topping, 1996):

1. Curriculum-based content, i.e., mentoring activities, is in line with course content.
2. The one-to-one relationship between the mentor and mentee.
3. Mentor preparation: a mentor is a senior person who is more skilled and has more advanced experience than the mentee.
4. Continuity: The roles of the mentor and the mentee need not be permanent.
5. Space: Mentoring sessions are conducted at their own location.
6. Time: Mentoring sessions are scheduled outside of a class.
7. Mentee characteristics: Although some group activities may be proposed, the mentoring addresses personal goals and projects.
8. Mentor characteristics: A mentor can establish a sustainable relationship based on personal care, emotional support, and nurturing. They are experts and guides who instruct, impart knowledge, and challenge novice protégés in a real context (Orland-Barak, 2014).
9. Objectives: Mentoring activities may target formal academic achievement, a wide range of personal gains, such as intellectual, affective, attitudinal, personal, social, and emotional gains, or any combination thereof.
10. Mentoring framework: A personal and professional relationship is established during an educational process; this systemic reform strategy builds human capacity (Mullen and Klimaitis, 2021).

### 1.3. Research hypotheses

This research aims to determine the importance of mentoring in higher education in Spain and to identify the differences between the universities that offer mentoring and accompaniment programs for their students.

The research hypothesis considered in this research is as follows:

1. Spanish universities provide some type of mentoring program for their students.
2. Mentoring programs address university students in their final years to facilitate their transition to their professional lives.
3. Mentors are more experienced students, teachers, or professionals who participate in university programs.

## 2. Materials and methods

The websites of different university institutions were analyzed, gathering information to compare the different types of accompaniment programs universities offer to their students and their specific characteristics.

The methodology of this study has the following stages: The first stage was to identify universities with mentoring programs. This search was conducted through the search option on the university's websites. Two keywords were used: “mentoring” and “accompaniment.” This made it possible to gather information, which is sometimes scarce, because no mention of these keywords appeared in the first search or the search led to publications. In other cases, the search led directly to the regulated programs or to the proposals being developed or already implemented. For this purpose, a table was created to be included as Supplementary material for this publication. To elaborate this table, the following features were considered: university name, autonomous community, type of university (public or private), whether or not it has a mentoring program, the name of the program, who the mentors are, if they are teachers or university personnel or students (usually in the final years of their degree) if the program is evaluated, and finally, to whom (students) the program is addressed.

This table compares universities with mentoring programs, plans, or models established throughout the university and/or in specific faculties. The second stage of this research was to conduct a detailed analysis of other features of these programs, their principal objectives, and their expected outcomes. The study analyzed whether mentoring is done individually or in groups, optional or within the curriculum, regulated or associated with a specific course, competence-based itinerary, and whether the program includes mandatory tasks with a final mark. The analysis also included the training required by the mentor and whether or not they have previous mentoring experience, the methodology used in the mentoring, and other questions, such as whether the acquisition of competencies is assessed, if the program and the mentor are evaluated, and, finally, the number of sessions within the program.

## 3. Results

The results of the study made it possible to generate a wide field of research since they provided several variables. All aspects related to the role of the mentor, including who they were and their training and/or qualifications for this role, were considered relevant. It was also interesting to address the study from the perspective of the proposed mentoring methodology, which can be individual, group, or peer-to-peer. Another area of study could be the mentoring process itself, and the different activities and tasks that students should develop. Finally, it was interesting to compare what was important and necessary for students who were in their first year at the university with those who were nearing graduation.

The initial results showed that, out of 60 universities, 42 were public, 18 were private, and only 17 did not offer students any mentoring program. Of these, 12 were public and 5 were private universities. Of the public universities, there was one autonomous community, Aragon, whose public university, the University of Zaragoza, had an accompaniment program on any of its three campuses. In Andalucía, the University of Huelva and the University of Malaga offered no programs. In the other seven autonomous communities that possess any university, there were seven universities without mentoring programs: Universitat de les Illes Balears (Baleares); Universidad María Zambrano UVA (Castilla y León); Universidad de Cantabria (Cantabria); Universidad de Alicante (Valenciana); Universidad de Alcalá (Madrid); Universidad Pública de Navarra (Navarra); and Universidad de Murcia (Murcia).

**Figure 1:**
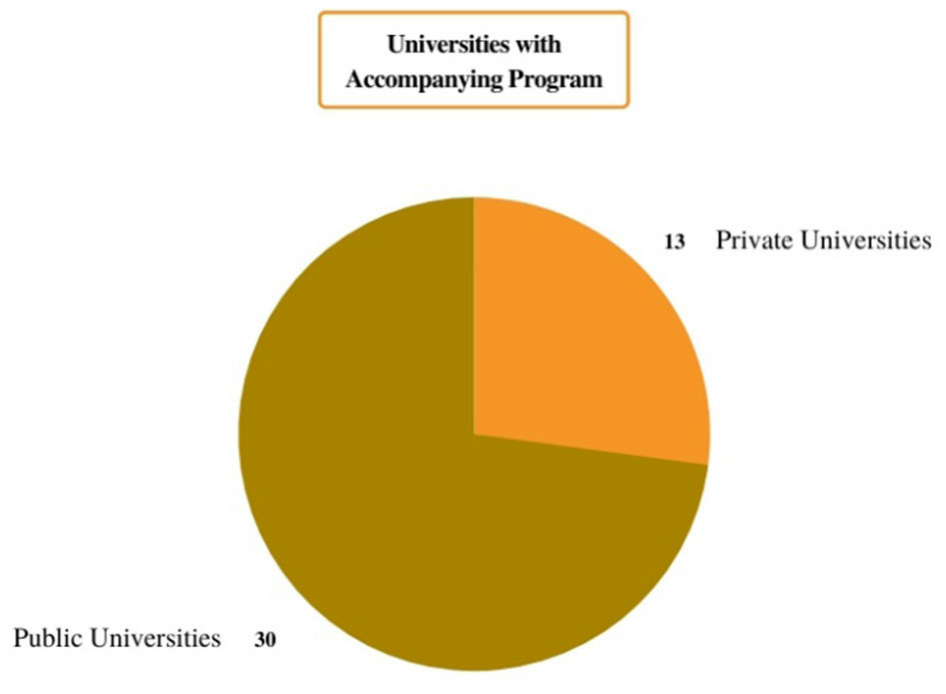
Graph of public and private universities with mentoring programs.

Among all the 43 universities that had a mentoring program, plan, or project, there were more than 54 mentoring programs, most of them unique to their institution (see Figure 1). Only three public universities (the University of Granada, the University of Seville, and Carlos III University) offer two programs each, and three private universities, the Universidad San Pablo CEU, the Francisco de Vitoria University (UFV), and the University of Navarra, have two, five, and four programs, respectively.

Madrid is the autonomous community with the highest number of universities, with a total of 14 universities, 10 of which are private, offering two or more regulated accompaniment programs. Of the four public universities in Madrid, the Universidad a Distancia (UNED), the Universidad Complutense de Madrid, the Universidad Politécnica, and the Universidad Carlos III, three offer mentoring or accompaniment programs in specific faculties rather than for all degree programs. Only at the Universidad Carlos III are mentor alumni with a minimum of 3 years of mentoring experience allowed. At other universities, teachers offered mentoring services as part of their professional duties and did not receive extra financial compensation.

Of the 54 Accompanying Programs, 29 of them involved a mentor who is a five-year student pursuing a degree. Most reminder programs (from 15 universities) had a teacher in charge of overseeing the mentoring process, with these activities being integrated into their teaching responsibilities. In four universities, two public (Universidad Carlos III and Universidad Politécnica de Cartagena) and two private (the CEU San Pablo University and the Pontifical University of Comillas, both in the Comunidad de Madrid), the programs are carried out by alumni. Moreover, UFV has a unique program in Spain with 264 mentors, of whom 83 were hired exclusively for mentoring tasks.

Across the rest of Spain, only three other private universities provide some form of accompaniment to their students: the Universitat Pompeu Fabra in Barcelona, the Universidad Católica San Antonio de Murcia, and the University of Navarra.

**Figure 2:**
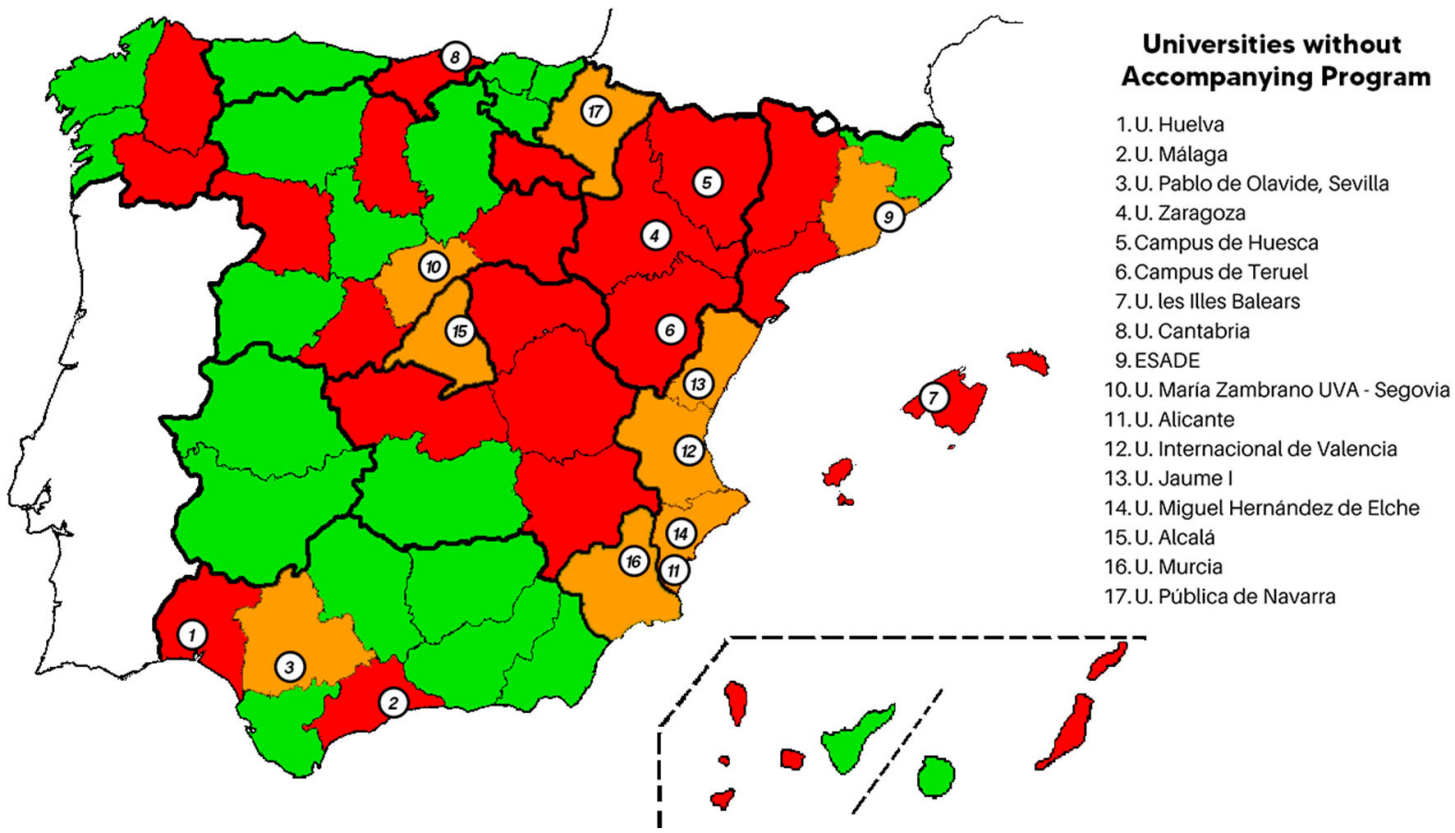
Distribution of universities and mentoring programs in Spain. Provinces with universities with mentoring programs are marked in green; those without mentoring programs are marked in red; and provinces with and without mentoring programs are marked in orange.

Other universities offering mentoring programs are located in all autonomous communities across Spain, from the one with the most universities in the community to the one with the fewest. Figure 2 shows the distribution of universities and mentoring programs in Spain.

Of all universities, four are polytechnic schools, of which three provide a mentoring program where mentors are final-year students and in the last one, the Polytechnic University of Cartagena, mentors are alumni. This particular mentoring program is aimed exclusively at students in their final year, while at the Polytechnic University of Catalonia and Madrid, the program is exclusively aimed at accompanying first-year students. The Polytechnic University of Valencia is the only one that accompanies students throughout their university studies.

Regarding the beneficiaries of the accompaniment programs, only 11 universities have programs aimed at all students, and of these universities, eight are private, and five are in Madrid. Notably, all these universities rely on academics, experts, and even qualified personnel to accompany students. Of the other three public universities, only one offers accompaniment provided by students in their final years.

Concerning universities that promote mentoring programs among first-year students, of the 23 universities, 19 are public. Moreover, four of them are incoming students, without distinction according to their year. There are two exceptional cases, such as the Universidad de Leon, which accompanies its students in the first and second years, and the Universidad de Navarra, which has a Core Curriculum program that accompanies students in the first, second, and third years. A total of 11 universities accompany all their students, regardless of the year. The remaining eight are situated in the community of Madrid, with the UFV again having a specific program according to the student's year of study.

At UFV, for first-year students, the program is linked to a subject common to all degrees called Skills and Competencies of the Person, which has a community part in the classroom and an individual part with six mentoring sessions. In the second year, again in all degrees, all students attend the “Education for Social Responsibility” course, divided into three areas: classroom, social practices, and mentoring sessions, two individual and one in groups (Queiruga-Dios, 2020). In these two courses, the mentor plays a fundamental role. In the second and third academic years, depending on the degree, students have the “Protagonize Your Future” program. Meaningful learning situations related to students' personal, academic, and professional orientations are designed (Backer, 2015). This program is currently being expanded to include more degrees. In addition, they offer another program for incoming students, which, as at four other public universities, is called the “Buddie program.” For third- and fourth-year students during their international experience, the “UFV Planet” program is available.

In the “Buddie Program,” 100% of mentors are students in their final years who offer to host those new students at their university. In the case of San Pablo CEU University, the “Tutorial Action” program is provided to first-year and international students with a professor as a mentor.

Only seven universities have programs to accompany students in their professional development, three of which are public (the University of Seville, the University of Salamanca, and the University of Carlos III) and four are private, all of which are located in the Community of Madrid (ESIC, CEU, UFV, and Universidad Pontificia de Comillas). All of these programs use the word “mentoring,” except for the UFV program called “Be the Protagonist of Your Future.”

At the University of Seville, the mentorship program involves both mentors who are teachers from the institution itself. However, in other universities, the mentoring work is carried out by alumni who have accumulated 3 to 15 years of experience. In the case of UFV, the mentor can be a professor or a professional hired for this purpose.

Six public universities (the University of Cadiz, the Polytechnic of Catalonia, the University of Valencia, the University of Carlos III, and the University of the Basque Country) and only one private one (the UFV) offer a peer mentoring program. The reality is that each university program has a different name. It should be noted that, in this program, a student accompanies another student, and this is not an evaluable action. In the case of students acting as mentors, their tasks are recognized with credits.

The University of Jaen is the only university that offers a mentoring program for researchers aimed exclusively at graduate students. The mentor in this program is a professor.

Although this analysis has focused on the type and characteristics of mentorship programs, additional information is also worth noting.

Of the 43 universities with mentoring programs, 25 mentoring tasks rely on third- and fourth-year students to provide mentoring or accompaniment to younger students. In the remaining 19 universities, mentoring is provided by professionals, 12 of whom are university teachers who develop their teaching activities in the classroom and also in their accompaniment sessions.

**Figure 3:**
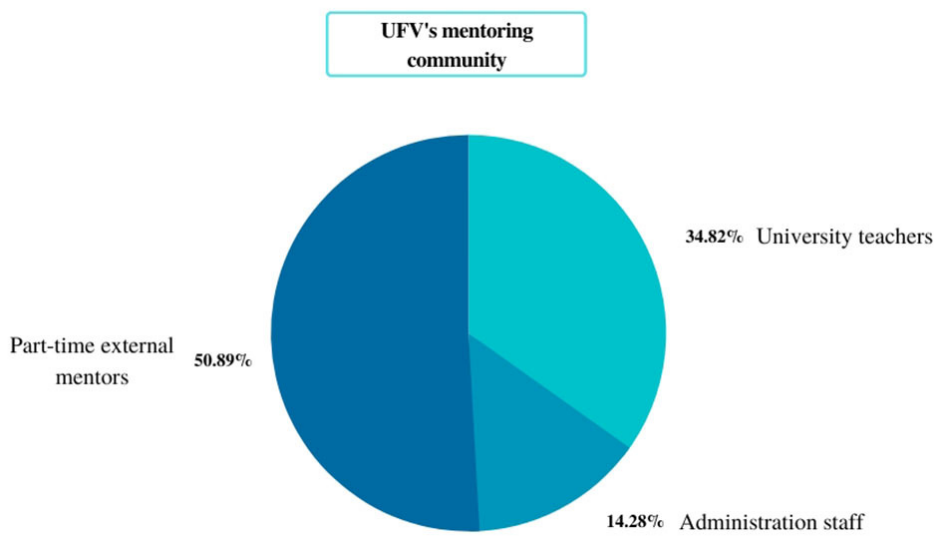
Graph with the distribution of mentor's profiles at UFV.

At UFV, there are three types of mentors: university teachers, university staff, and external mentors. During the 2022–23 academic year, a total of 224 mentors are employed by the UFV, 78 of whom are teachers; 32 are university staff with specific training to perform this function, while the rest are professional mentors hired part-time, depending on the number of students (see Figure 3). In addition to mentoring, these external professionals also perform other tasks while dedicating a number of hours per week to accompanying students.

Another aspect that has been measured in this comparative study is whether the accompaniment program is linked to any specific course. This was not the case in all universities, except for one program at the University of Navarra: “Core Curriculum,” a mandatory course accounting for 18 ECTS, and the programs implemented at the UFV.

Specifically, at UFV, the program accompanying first-year students is closely linked to the course of Skills and Competencies of the Person (Queiruga-Dios et al., 2023). This course is taught with the same name in several degree programs, while in others, it may have a slightly different name that is specifically associated with the respective degree program. In the second year, the accompaniment program goes hand in hand with two other areas (classroom, social action, and accompaniment) taught in Education for Social Responsibility. In the degrees where the “Be the Protagonist of Your Future” the program is implemented in the third and fourth years, the link to the degree program is more varied and almost always part of curricular practices. The only exceptions to the UFV-regulated programs where the accompaniment program is not linked to a subject are the peer accompaniment program carried out by the Allies Academy and the UFV Planet program, which is an optional accompaniment program for students who are going to do an off-campus international experience or those who come from other universities.

Another notable aspect is the little or no specific training required by those who provide support, especially teachers. In mentoring programs where the mentor is a final-year student, no previous mentoring experience is required, but they are offered some training to perform this task. In most cases, the training is summarized in 15 to 20 h.

At UFV, however, mentor training is permanent and constant. Mentors are selected and provided with an intensive course of 20 h and 6 h of individual accompaniment, where they experience the same type of mentoring they will also provide (Law et al., 2020; Stelter et al., 2021). Two-day mentor training sessions are held annually for the entire mentoring community, with asynchronous training modules that can constantly return. Furthermore, a mentor-trainer accompanies mentors throughout the academic year, with additional training in the sciences more associated with accompaniment, including anthropology, psychology, pedagogy, and so on.

## 4. Discussion

One of the results of this research is that the mentoring processes within universities are becoming increasingly prevalent. More and more institutions are concerned about this issue. However, the authors found that not many studies have highlighted the value of accompaniment, and even fewer have conducted comparative analyses of the diverse realities across various universities. Mentoring programs will have the potential to be part of a university's strategy to help students succeed when the shortcomings of mentoring research are addressed. In response to the large number of students who fail to graduate and drop out of the university, colleges and universities have created mentoring programs to help students succeed. Many colleges and universities have established mentoring programs to help students succeed. There is a wide variation in the structure of mentoring programs, such as who acts as a mentor (e.g., faculty, peers, alums, and so on), the level of training of mentors (formal and informal), the theoretical underpinnings (e.g., mentoring theory), and the structure of mentoring programs (Law et al., 2020).

There is almost one model and method per university, but young Spanish university students are the same in Galicia as in Murcia. In terms of psychological traits, levels of experience, and their historical environment, they all face the same questions, such as, Have I chosen the right degree? or Will I find a job when I finish my studies? Given this situation, it is important to determine the best way to accompany these students and provide them with meaningful learning experiences and environments that allow them to fulfill their personal, academic, and professional goals.

As the comparative study shows, only seven universities have mentoring programs offering students accompaniment and guidance for their oriented professional futures. However, the rest of the universities dedicate their efforts to accompanying students in their initial contact with university life.

Efforts are being made to implement mentoring and accompaniment programs within higher education. However, it is also necessary to provide guidance and accompaniment both before the start of university life and throughout students' university experiences. Once students start university studies, it is important to accompany them and guide them in their choices of studies, encouraging them to engage with important questions about their lives and futures.

Mentoring programs at universities must begin with the adequate and systematic training of mentors. This training should not be limited to external and formal mentoring that will not provide support to students (Jiang et al., 2022). Mentors accompany, advice, and guide their students, and they are references to the student's life and have an impact on the student's future professional life. Therefore, gathering specific qualities and competencies such as design, planning, organization, and evaluation will facilitate the process of integral student development (Ogbuanya and Chukwuedo, 2017).

Nora and Crisp (2007) theoretically framed the underlying components that students identified as constituting a mentoring experience. They identified four major domains or latent constructs from the mentoring literature:

- Psychological/emotional support: listening, providing moral support, identifying problems, and providing encouragement
- Goal setting and career paths: assistance in academic and career goals and decision-making
- Academic knowledge support: acquisition of necessary skills and knowledge to change mentees academically.
- Role model: the mentee's ability to learn from a mentor's present and past actions (achievements and failures).

## 5. Conclusion

The number of accompaniment and mentoring programs offered by Spanish universities continues to rise.

This research aimed to determine the importance of accompaniment and mentoring in Spanish universities, some of which offer different and specific mentoring programs designed to enhance and further the kind of education and preparation institutions of higher learning should ideally provide.

Accompaniment processes generally have a longer duration in private universities than in public universities, offering a wider range of programs for both current and incoming students and those with specific needs, such as international students. This study opens new avenues for research into the ideal profile of mentors to best accompany university students.

This study found that, out of the 71 current mentoring programs, 30 mentors are senior students who share their lived experiences of university life. However, student mentors cannot provide the richer advice and guidance that comes with years of maturity and professional work (Ogbuanya and Chukwuedo, 2017; Okolie et al., 2020). Lectures play a key role in providing career advice. The current labor market suggests that newcomers to a professional job need several attributes, skills, and competencies that may change over time and in different contexts. New workers need to be connected with their surrounding context (Donald et al., 2018; Monteiro et al., 2022).

This study also shows how little specific training or experience is required by those providing mentoring and accompaniment and how very few programs have a regulated itinerary that serves as a guide for mentoring.

In the comparative study of Spanish universities, the main objective of the accompaniment programs is to help first-year students integrate into their university academic life and to understand the facilities and areas that will allow them to develop in the fields that interest them most. The only case that is out of the ordinary is the one presented by UFV, with a program developed in interpersonal and intrapersonal competencies that allows students to ask themselves the most fundamental questions. They follow a path that first takes place in six individual meetings with their mentor and is complemented by the subject of the person's Skills and Competencies.

One of the limitations of this study was the lack of information on the web pages of the universities, which are not updated. Not all of them have updated or organized information about their mentoring programs, making it difficult to make a completely reliable comparison.

## Data availability statement

The original contributions presented in the study are included in the article/Supplementary material, further inquiries can be directed to the corresponding author.

## Author contributions

SG-C and MQ-D contributed to the conception and design of the study. SG-C collected the data, prepared the database, and wrote the first draft of the manuscript. AQ-D and MQ-D reviewed the manuscript and completed it. All authors contributed to the manuscript revision, read, and approved the submitted version.
