# Supplementary material for: Comparative study of accompaniment programs for undergraduate degree students in Spanish universities
Source: Front Psychol. 2023 Jun 23;14:1165232. doi: 10.3389/fpsyg.2023.1165232 (PMC10326380; doi:10.3389/fpsyg.2023.1165232)
Supplement: Supplementary file 1 [file Table_1.docx]

Supplementary Material

Comparative study of accompanying programs for undergraduate degree students in Spanish universities

Susana García-Cardo, Marián Queiruga-Dios, Araceli Queiruga-Dios

*** Correspondence:** Corresponding Author: queirugadios@usal.es

# Data collected during the study

The Table included here provides the information collected during the comparative analysis that was developed. The information provided is the name of the university, the location (autonomous community or Spanish region), the type of institution (public or private), the program name (in case it has), who acts as mentor (a teacher or a student), if the mentoring program includes any kind of evaluation or mark, and finally, the stakeholder of the program.

| **University** | **Autonomous community** | **Type** | **Program name** | **Mentor** | **Mark** | **Address to** |
| --- | --- | --- | --- | --- | --- | --- |
| De Almería | Andalucía | Public | Mentor Buddy Program | Student | No | Foreign students |
| De Cádiz | Andalucía | Public | Colleague project | Student | No | First-year students |
| De Córdoba | Andalucía | Public | Tutorial action plan | Student | No | First-year students |
| De Granada | Andalucía | Public | Mentor Program | Student | No | First-year students |
|  | Andalucía |  | Buddy Program | Student | No | Foreign students |
| De Huelva | Andalucía | Public |  |  |  |  |
| De Jaén | Andalucía | Public | Mentoring program for researchers | Teacher |  | Postgraduate students |
| De Málaga | Andalucía | Public |  |  |  |  |
| De Sevilla | Andalucía | Public | Writing mentoring | Teacher and student | No | First-year and final term project students |
|  | Andalucía |  | Mentoring program | Student | No |  |
| De Pablo de Olavide, Sevilla | Andalucía | Private |  |  |  |  |
| De Zaragoza | Aragón | Public |  |  |  |  |
| Campus de Huesca | Aragón | Public |  |  |  |  |
| Campus de Teruel | Aragón | Public |  |  |  |  |
| De Oviedo | Asturias | Public | Tutorial action plan | Student | No | First-year students |
| De les Illes Balears | Baleares | Public |  |  |  |  |
| De La Laguna | Canarias | Public | Orientation and tutorial action plan | Teacher | No | All students |
| De las Palmas de Gran Canaria | Canarias | Public | Buddy program | Student | No | Foreign students |
| De Cantabria | Cantabria | Public |  |  |  |  |
| ESADE | Cataluña | Private |  |  |  |  |
| Autònoma de Barcelona | Cataluña | Public | Mentoring plan | Student | No | First-year students |
| De Barcelona | Cataluña | Public | Training in competencies |  |  |  |
| De Girona | Cataluña | Public | Mentoring plan | Student | No | First-year students |
| Politécnica de Catalunya | Cataluña | Public | Peer mentoring | Student | No | First-year students |
| Pompeu Fabra, Barcelona | Cataluña | Private | Mentoring Program | Student |  | First-year students |
| De Castilla-La Mancha | Castilla - La Mancha | Public | Mentor program | Student | No | First-year students |
| De Burgos | Castilla - León | Public | Mentor program | Student | No | First-year students |
| De León | Castilla - León | Public | Tutorial action plan | Teacher | No | First and second-year students |
| De Salamanca | Castilla - León | Public | Mentor program | Teacher | No | Third and fourth-year students |
| María Zambrano UVA - Segovia | Castilla - León | Public |  |  |  |  |
| De Valladolid | Castilla - León | Public | Mentor program | Student | No | First-year students |
| De Alicante | C. Valenciana | Public |  |  |  |  |
| Internacional de Valencia | C. Valenciana | Private |  |  |  |  |
| Jaume I | C. Valenciana | Private |  |  |  |  |
| Miguel Hernández de Elche | C. Valenciana | Private |  |  |  |  |
| Politécnica de Valencia | C. Valenciana | Public | Comprehensive student accompaniment plan | Teacher and student | No | All students |
| De Valencia | C. Valenciana | Public | Peer mentoring | Student | No | First-year students |
| De Extremadura | Extremadura | Public | Buddy program | Student | No | Foreign students |
| De Vigo | Galicia | Public | Tutorial action plan | Student | No | Incoming students |
| Santiago de Compostela | Galicia | Public | Mentoring project. Professional growth | Student | No | All students |
| Da Coruña | Galicia | Public | PAT – Tutoring and mentoring | Student | No | Incoming students |
| ESIC | Madrid | Private | Mentoring | Coach | No | Senior and recent graduate students |
| IE University | Madrid | Private | Mentorship society | Student | No | First-year students |
| UNED | Madrid | Public | Mentoring program | Student | No | First-year students |
| De Alcalá | Madrid | Public |  |  |  |  |
| Alfonso X | Madrid | Private | Tutor | Teacher and staff | No | All students |
| Camilo José Cela | Madrid | Private | Mentoring program | An expert | No | All students |
| Carlos III | Madrid | Public | Mentoring program | Alumni | No | Senior and recent graduate students |
|  | Madrid |  | Colleagues program | Student | No | First-year students |
| CEU San Pablo | Madrid | Private | Tutorial action | Teacher | No | First-year and foreign students |
|  | Madrid |  | Mentoring | Alumni | No | Alumnos último curso /recién graduado |
| Complutense de Madrid | Madrid | Public | Mentoring program | Student | No | First-year students |
| Europea de Madrid | Madrid | Private | Academic advisor | Staff | No | All students |
| Francisco de Vitoria | Madrid | Private | Personal skills and competences | Teacher | Yes | First-year students |
|  | Madrid |  | Social Responsibility | Teacher | Yes | Second-year students |
|  | Madrid |  | UFV Planet | Teacher | No | Foreign students |
|  | Madrid |  | *Protagoniza tu futuro* [Protagonize your future] | Teacher | Yes | Third and fourth-year students |
|  | Madrid |  | Partners academy | Student | No | All students |
| Nebrija | Madrid | Private | Office of Psychopedagogical Guidance | Expert | No | All students |
| Politécnica de Madrid | Madrid | Public | Mentoring and tutoring program Tutela | Student | No | First-year students |
| Pontificia de Comillas | Madrid | Private | Programa Mentoring | Alumni | No | Third and fourth-year students |
| Villanueva | Madrid | Private | Tutoring program | Teacher | No | All students |
| Católica San Antonio de Murcia | Murcia | Private | Personal tutoring | Teacher | No | All students |
| De Murcia | Murcia | Public |  |  |  |  |
| Politécnica de Cartagena | Murcia | Public | Mentoring program | Alumni | No | Senior students |
| De Navarra | Navarra | Private | Mentoring | Teacher and staff | No | All students |
|  | Navarra |  | Buddy Program | Student | No | Incoming students |
|  | Navarra |  | Core curriculum courses | Teacher | Yes | First, second and third-year students |
|  | Navarra |  | Guidance | Counselor | No | Students applying (before University) |
| Pública de Navarra | Navarra | Public |  |  |  |  |
| De País Vasco | País Vasco | Public | Peer mentoring | Student | No | Incoming students |
